# Chemical Composition and In Vitro and In Silico Antileishmanial Evaluation of the Essential Oil from *Croton linearis* Jacq. Stems

**DOI:** 10.3390/antibiotics11121712

**Published:** 2022-11-28

**Authors:** Jesús García-Díaz, Julio César Escalona-Arranz, Ania Ochoa-Pacheco, Sócrates Golzio Dos Santos, Rosalia González-Fernández, Julio Alberto Rojas-Vargas, Lianet Monzote, William N. Setzer

**Affiliations:** 1 Pharmacy Department, Natural and Exacts Sciences Faculty, Oriente University, Avenida Patricio Lumumba s/n, Santiago de Cuba 90500, Cuba; 2 Department of Pharmaceutical Sciences, Federal University of Paraiba, João Pessoa 58059-900, Brazil; 3 Toxicology and Biomedicine Centre (TOXIMED), University of Medical Science, Autopista Nacional Km 1½ s/n, Santiago de Cuba 90400, Cuba; 4 Chemistry Department, Natural and Exacts Sciences Faculty, Oriente University, Avenida Patricio Lumumba s/n, Santiago de Cuba 90500, Cuba; 5 Department of Parasitology, Institute of Tropical Medicine “Pedro Kourí”, Apartado Postal No. 601, Marianao 13, La Habana 10400, Cuba; 6 Research Network Natural Products against Neglected Diseases (ResNetNPND); 7 Department of Chemistry, University of Alabama in Huntsville, Huntsville, AL 35899, USA; 8 Aromatic Plant Research Center, 230 N 1200 E, Suite 100, Lehi, UT 84043, USA

**Keywords:** essential oil, *Croton linearis*, *Leishmania amazonensis*, *epi*-*γ*-eudesmol

## Abstract

*Croton linearis* Jacq. is an aromatic shrub that has been utilized in traditional medicine in the Bahamas, Jamaica, and Cuba. Recent studies have revealed the antiprotozoal potential of its leaves. The present work is aimed to identify the volatile constituents of essential oil from the stems of *C. linearis* (CLS-EO) and evaluate its in vitro antileishmanial activity. In addition, an in silico study of the molecular interactions was performed using molecular docking. A gas chromatographic–mass spectrometric analysis of CLS-EO identified 1,8-cineole (27.8%), *α*-pinene (11.1%), *cis*-sabinene (8.1%), *p*-cymene (5.7%), *α*-terpineol (4.4%), *epi*-*γ*-eudesmol (4.2%), linalool (3.9%), and terpinen-4-ol (2.6%) as major constituents. The evaluation of antileishmanial activity showed that CLS-EO has good activity on both parasite forms (IC_50Promastigote_ = 21.4 ± 0.1 μg/mL; IC_50Amastigote_ = 18.9 ± 0.3 μg/mL), with a CC_50_ of 49.0 ± 5.0 μg/mL on peritoneal macrophages from BALB/c mice (selectivity index = 2 and 3 using the promastigote and amastigote results). Molecular docking showed good binding of *epi*-*γ*-eudesmol with different target enzymes of *Leishmania*. This study is the first report of the chemical composition and anti-*Leishmania* evaluation of CLS-EO. These findings provide support for further studies of the antileishmanial effect of this product.

## 1. Introduction

Neglected tropical diseases (NTDs) are a major public problem in the health systems of many countries, causing significant morbidity and mortality. Among these, leishmaniasis is a parasitic disease caused by an obligate intracellular parasite of genus *Leishmania*, which is transmitted to humans by the bite of infected female phlebotomine sandflies. This disease has wide clinical spectra, with three main forms being recognized: visceral (also known as *kala-azar*, which is the most severe form of the disease), cutaneous (the most common), and mucocutaneous [1,2]. 

Approximately 350 million people in 98 countries are at risk of infection, and it is estimated that 700,000 to 1 million new cases occur annually and between 20,000 and 30,000 deaths occur each year [3,4]. Currently, vaccines are not yet available, and the conventional treatments constitute the main weapon to control the diseases. However, the available commercial drugs, namely pentavalent antimonials, amphotericin B and lipid formulations, pentamidine, miltefosine, and paromomycin, display several drawbacks, including severe adverse effects, teratogenicity, parenteral administration, and prohibitive costs. In addition, the resistance of the parasite to the clinically used drugs has emerged in endemic areas [5,6]. For these reasons, there is an urgent need to research new and more effective drugs to replace or supplement the present therapy. 

In this sense, medicinal plant extracts and their metabolites are likely to provide a valuable source of new antileishmanial agents [7,8,9]. Among natural products, essential oils (EOs) and their pure components have shown remarkable in vitro and in vivo activities against different species of *Leishmania* [10,11]. The lipophilic property of EOs and their constituents permits easy diffusion across cell membranes and interactions with different intracellular targets of the protozoa. The literature reports several mechanisms for both forms of *Leishmania* spp. for EOs and their terpene components, e.g., affect permeability of cell membranes, the inhibition of cellular isoprenoid biosynthesis, the disruption of specific metabolic pathways of lipids or proteins, the depolarization of mitochondrial membranes, and the stimulation of microbicidal mechanisms such as NO production [12,13,14].

The *Croton* genus is one the most representative of the Euphorbiaceae, with a wide diversity of active metabolites, mainly alkaloids, phenolic compounds, and terpenoids [15,16,17,18]. Some *Croton* spp. produce EOs, and their biological potentials have been demonstrated [15,19,20,21,22]. In Cuba, 47 species of *Croton* have been described, with some of them used in folk medicine by the population to treat several ailments [23]. However, there are scarce studies about the phytochemical and biological potentialities of these species, which has limited their medicinal value and utility [24,25,26]. Among these species, *Croton linearis* Jacq. has been studied by our research group, having reported the antimicrobial and antiprotozoal effects of extracts and isolated compounds (mainly alkaloids) from leaves [27]. In addition, the chemical composition and in vitro antileishmanial activity of EOs obtained from leaves was reported, demonstrating good results on the promastigote (IC_50_ = 20.0 ± 4.9 µg/mL) and amastigote (IC_50_ = 13.8 ± 4.3 µg/mL) forms [28]. However, ethnomedical information about the applications of this plant against leishmaniasis or other skin diseases was not found. Nevertheless, some species of *Croton* have traditionally been used. For example, *Croton roxburgii* N.P.Balakr and *Croton sublyratus* Kurz. have been used as herbal treatments for skin diseases and other ailments, and their inhibition of melanin content was demonstrated [29]. On the other hand, *Croton adamantinus* Müll.Arg. [30] and *Croton sylvaticus* Hochst. [31], used on skin wounds and dermatological pathologies, displayed anthelmintic effects and antioxidant activity, respectively.

Considering the antiprotozoal potential observed for the *Croton* genus and, in particular, the leaves of this species, the purpose of this work was to determine the chemical composition of the EO of stems from *C. linearis* (CLS-EO) and to evaluate its effect on *Leishmania* spp. as a continuation of our on-going investigations. Additionally, a molecular docking analysis aimed to clarify the behavior of the major components present in the EO in the antileishmanial activity. Different enzymes were chosen because they play roles in the metabolic pathways of parasites and are considered potential targets for the development of new antiparasitic compounds [32].

## 2. Results

### 2.1. Extraction and Essential Oil Analysis

**Figure 1:**
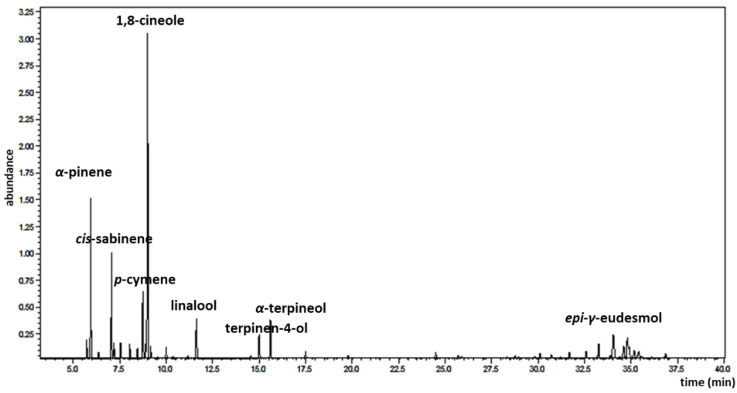
Gas chromatography–mass spectrometry (GC-MS) profile of *Croton linearis* stem essential oil growing in the Siboney-Juticí Ecological Reserve, Santiago de Cuba (major components are shown).

The CLS-EO obtained by a hydrodistillation–cohobation method using a Clevenger-type apparatus presented a yield of 0.9% (*v*/*w*). The GC/MS analysis revealed a total of 56 volatile compounds in the CLS-EO, representing more than 99% of the total peak area. A representative gas chromatographic profile of the CLS-EO is shown in Figure 1, while the mass spectra of the main compounds are available in the Supplementary Material (Figure S1).

**Figure 2:**
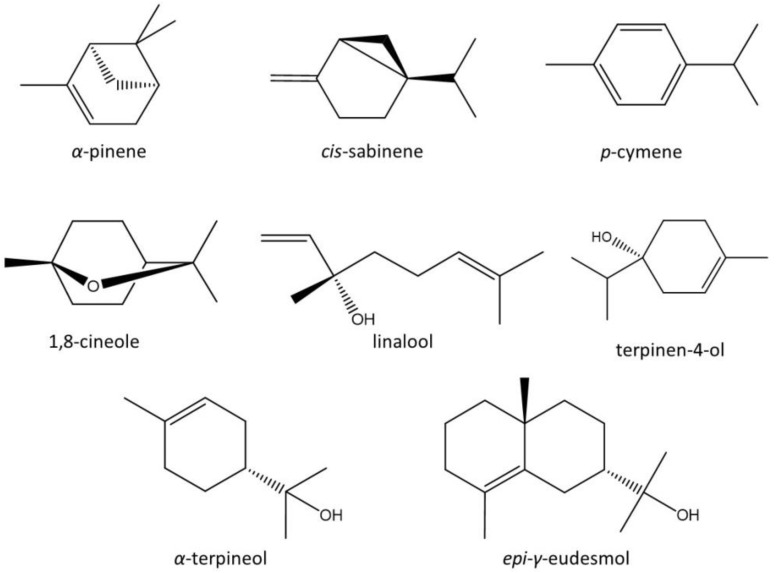
Chemical structures of the main compounds identified in the essential oil of stems from *Croton linearis* growing in the Siboney-Juticí Ecological Reserve, Santiago de Cuba.

In the studied oil, all compounds were of a terpenoid nature, including 26 monoterpenes and 31 sesquiterpenes (Table 1). The main constituents of CLS-EO were 1,8-cineole (27.83%), *cis*-sabinene (8.06%), *α*-pinene (11.05%), *p*-cymene (5.72%), *α*-terpineol (4.35%), *epi*-*γ*-eudesmol (4.15%), linalool (3.91%), and terpinen-4-ol (2.55%) (Table 1, Figure 2).

### 2.2. Antileishmanial Activity and Cytotoxicity

In the present study, the effect of CLS-EO was evaluated against the promastigote and amastigote forms of *L. amazonensis* for the first time. Additionally, the cytotoxicity was assayed on peritoneal macrophages from BALB/c mice. CLS-EO showed good inhibitory activity against both parasite forms of *L. amazonensis,* with IC_50_ values of 21.4 ± 0.1 µg/mL and 18.9 ± 0.3 µg/mL, respectively (Table 2). However, CLS-EO exhibited toxic effects on peritoneal macrophages from BALB/c mice, and the calculated SI indicates low selectivity of this product (Table 2).

### 2.3. Molecular Docking

After the in vitro evaluation, a molecular docking study was performed, aiming to predict the effects of the main compounds of CLS-EO (Figure 2) on several important enzymatic targets of *Leishmania*. The docked poses for each compound were evaluated, selecting the ones with the lowest ΔG_dock_ (Table 3). After docking score normalization to account for molecular weights was carried out, trypanothione synthetase (TryS) and 14α demethylase (Cyp51) stood out as the protein targets most susceptible (DS_norm_ < −6 kcal/mol) to the CLS-EO compounds. Table 3 also highlights *epi*-*γ*-eudesmol as the most potentially active metabolite, which presented better docking parameters for more than one target. In particular, for TryS and ArgI, the docking scores were superior to the commercial antileishmanial drugs used as standards.

In addition to *epi*-*γ*-eudesmol, some other CLS-EO metabolites showed docking affinities to the *Leishmania* target enzymes. These included terpinen-4-ol, 1,8-cineole, and α-pinene for Cyp51 and *cis*-sabinene, α-pinene, terpinen-4-ol, linalool, and *p*-cymene for trypanothione reductase (TryR). As declared before, TryS was the best target for CLS-EO metabolites, with all main compounds presenting DS_norm_ values lower than −6 kcal/mol, except for 1,8-cineole. In addition, α-terpineol, linalool, and *epi*-*γ*-eudesmol docked well to ArgI (Table 3).

**Figure 3:**
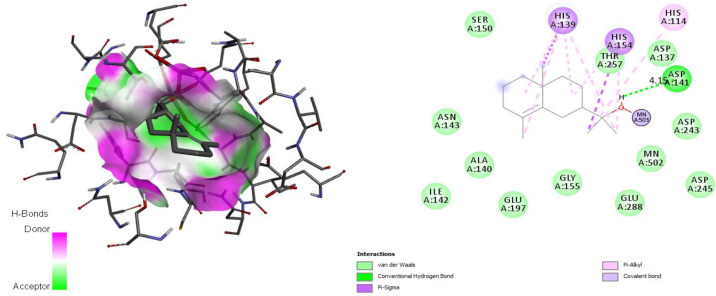
Interaction between *epi*-*γ*-eudesmol and arginase I, characterizing the amino acid residues of the catalytic site involved in the complex stabilization.

The docking simulation of the ArgI-ligand (*epi*-*γ*-eudesmol) complexes exhibited an H-bonding interaction with Asp141 in a radius of 4.15 Å. This interaction with Asp141 occupies the same position as Asp128, which is responsible for coordinating the Mn^2+^ cofactor in the active site [33]. As shown in Figure 3, the oxygen atom of the hydroxyl group engaged in coordination with a Mn^2+^ ion.

**Figure 4:**
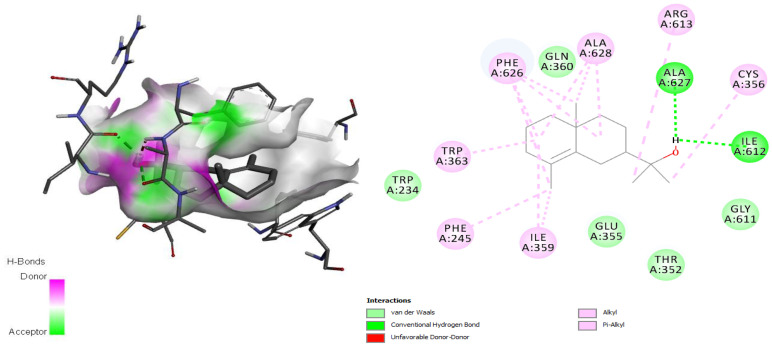
Interaction between *epi*-*γ*-eudesmol and the trypanothione synthetase target, characterizing the amino acid residues of the catalytic site involved in the complex stabilization.

**Figure 5:**
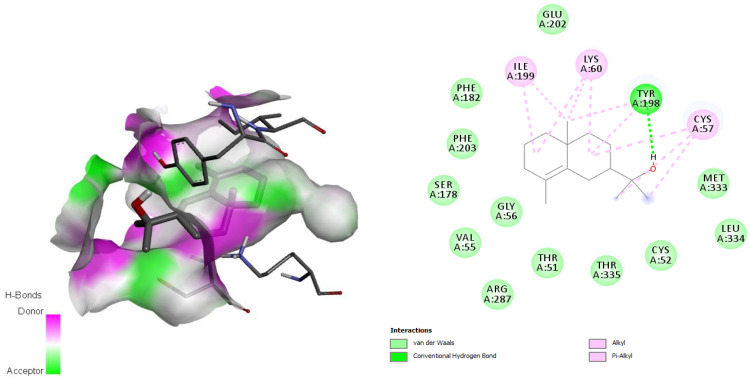
Interaction between *epi*-*γ*-eudesmol and the trypanothione reductase target, characterizing the amino acid residues of the catalytic site involved in the complex stabilization.

The TryS-ligand (*epi*-*γ*-eudesmol) complex showed hydrogen bonding interactions with residues Ile612 and Ala627 through the oxygen atom of the hydroxyl group present in *epi*-*γ*-eudesmol (Figure 4), in addition to many hydrophobic interactions involving different amino acid residues, including Gln360, Gly611, Glu355, Thr352 Cys356, Phe626, Ala628, Ile359, Phe245, Trp363, and Ala627, present in the active site [34]. The TryR-ligand complex also showed hydrogen bonds through the hydroxyl group in the ligand and the Tyr198 residue in the enzyme (Figure 5), and hydrophobic interactions, mainly van der Waals interactions, with the residues Glu202, Met333, Leu334, Cys52, Thr335, Thr51, Arg287, Cys57, Gly56, Val55, Ser178, Phe203, and Phe182, which are involved in the active site [35].

**Figure 6:**
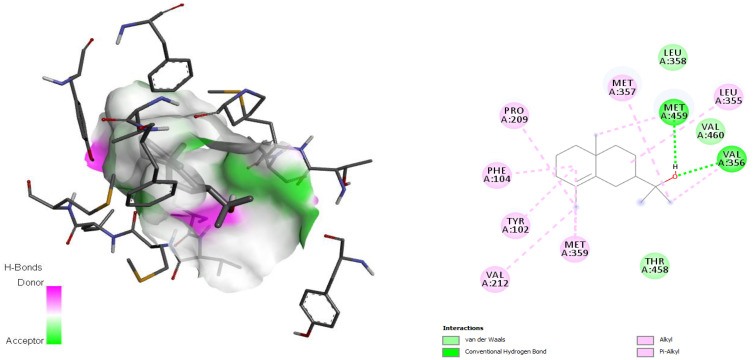
Interaction between *epi*-*γ*-eudesmol and the sterol 14*α* demethylase (Cyp51) target, characterizing the amino acid residues of the catalytic site involved in the complex stabilization.

According to Hargrove et al. in 2011 [36], the active site involved the amino acids Leu355, Met357, Leu358, Met459, Val212, Phe104, Met105, Tyr115, Ala114, Phe109, Gly282, Met283, Phe289, Leu129, Ala290, and Val460, and *epi*-*γ*-eudesmol was found to establish hydrogen bonds with Met459 and Val356, both involved in the active site. Moreover, for this complex, van der Waals and alkyl interactions with the amino acid residues Met357, Leu358, Leu355, Val 460, Val356, Pro209, Phe104, Tyr102, Met359, Thr458, and Val212 were observed (Figure 6).

## 3. Discussion

The studied CLS-EO showed a yield higher than other EOs from stems of *Croton* species, including *Croton heliotropiifolius* Kunth (0.17%) [21], *Croton rhamnifolioides* Pax & Hoffm (<0.1%) [37], *Croton pullei* Lanj. (0.06%) [38], *Croton pulegioides* Müll.Arg. (0.75%), *Croton rhamnifolius* var. *heliotropiifolius* (Kunth) Müll.Arg (0.01%) [39], and *Croton grewioides* Baill. (0.5%) [40].

With respect to the chemical analyses, this study is the first report of the composition of CLS-EO. However, a high variability could be appreciated in the main components of EOs extracted from stems of *Croton* spp. For example, Miranda et al. in 2019 [41] stated that the constituents of the EO from *Croton tricolor* Klotzsch ex Baill. were *epi*-globulol (19.0%), *α*-bisabolol (16.5%), *trans*-*α*-bergamotol (14.4%), and *β*-caryophyllene (9.1%). This last compound was the main constituent in EO from *C. rhamnifolioides* [37]. On the other hand, *C. grewioides* stems produced an oil with a predominance of phenylpropanoid compounds, the main components of which were (*E*)-anethole (47.8%) and (*E*)-methyl isoeugenol (30.0%) [40]. In 2007, Setzer et al. reported that the major components found in *Croton draco* Cham. & Schldl. bark essential oil were *β*-caryophyllene (31.9%), caryophyllene oxide (22.0%), 1,8-cineole (6.2%), and *α*-humulene (5.6%) [42].

In a 2011 study, Neves and da Camara [43] reported the chemical compositions of four EOs obtained from stems of *Croton* spp. The major identified components were *Z*-*α*-atlantone (24.3%) and *trans*-isolongifolanone (22.8%) in *Croton jacobinensis* Baill., camphor (16.6%) and tricyclene (12.8%) in *Croton rhamnifolius* (Baill.) Müll. Arg., *α*-bulnesene (32.9%) and guaiol (17.9%) in *Croton micans* (Sw.) Müll. Arg., and foenicolin (72.7%) in *Croton muscicapa* Müll. Arg.

Among the major components identified in CLS-EO, only *α*-pinene coincides with those reported for other species of the genus, such as *Croton antanosiensis* Leandri, *Croton adenocalyx* A. DC., *Croton argyrophylloides* Muell. Arg., *Croton zambesicus* Muell. Arg., and *Croton. sakamaliensis* Leandri; it is a compound that is considered, together with *β*-caryophyllene and *β*-pinene, to be a chemotaxonomic marker [44].

The chemical composition of the CLS-EO described in this study is very similar to the EO of leaves reported by Amado et al. in 2020 [45], with 48 compounds being found in both oils. The % relative abundance presented a comparable chemical profile, although there were differences observed in some compounds according to plant tissue, such as *α*-pinene (stems 11.05% vs. leaves 1.52%), *p*-cymene (leaves 3.37% vs. stems 5.72%), *β*-elemene (leaves 2.75% vs. stems 0.66%), *epi*-*γ*-eudesmol (leaves 2.75% vs. stems 0.66%), and hinesol (leaves 5.65% vs. stems 2.98%). In contrast, noticeable differences were observed with respect to other reports of leaf oils collected in the same geographical location [28]. These findings could be related to rainfall and other climatic characteristics of the habitat where this species grows (coastal xeromorphic scrub). Comparing the results with reports in the literature, differences and similarities among the EOs of leaves and stems from *Croton* species have been observed [37,39,44,46]. The differences in oil content and composition may be attributed to several factors, such as physiological variations, environmental conditions (climate, pollution, diseases, pests, and edaphic factors), geographic variation, genetic factors, season and harvest period, and others [47,48]. In this sense, it would be interesting, in future studies, to evaluate the effects of environmental parameters on the quality of EOs from leaves and stems of *C. linearis* growing in the Siboney-Juticí Ecological Reserve with prospects for future standardization.

Previously, we reported the antileishmanial activity of the EO from leaves of *C. linearis*, which was also active on both forms of *L. amazonensis,* with similar IC_50_ values: IC_50Promastigote_ = 20.0 µg/mL and IC_50Amastigote_ = 13.8 µg/mL [28]. Other *Croton* species have shown activity on *Leishmania* spp. EOs from *C. argyrophylloides*, *C. jacobinensis*, *Croton nepetifolius* Baill., and *Croton sincorensis* Mart. displayed activity against promastigotes of *L. chagasi*, *L. braziliensis,* and *L. amazonensis,* with an IC_50_ range between 9.1 and 27.0 μg/mL [49]. The EO from berries of *Croton macrostachyus* Hochst. ex Del. was effective against *L. donovani* and *L. aethiopica* promastigotes (IC_50_ = 0.1 μL/mL and 0.2 μL/mL, respectively) and the axenic amastigote stages (IC_50_ = 20.0 nL/mL and 6.66 nL/mL, respectively) [50]. *Croton cajucara* Benth leaf essential oil and its purified component 7-hydroxycalamenene showed in vitro activity against *L. chagasi* promastigotes, with IC_50_ values of 66.7 μg/mL and 11.4 μg/mL, respectively [20]. Thus, the potential of the *Croton* genus as a source of metabolites with antileishmanial effects could be highly suggested.

However, the observed effects of CLS-EO on the parasite could not be correlated with its main component, 1,8-cineole (27.8%), since the in silico docking parameters for this compound were not encouraging for three of the four targets that were modeled, being in agreement with experimental low antileishmanial activity against *L. amazonensis* (IC_50_ = 68.3 µg/mL) [51] or other species of *Leishmania* [11,52]. Nevertheless, other components with lower relative abundance have been described as active, for example, linalool (3.9%, IC_50Promastigote_= 4.3 ng/mL and IC_50Amastigote_ = 4.4 ng/mL) [53], *α*-pinene (11.1%, IC_50Promastigote_= 19.7 µg/mL and IC_50Amastigote_= 15.6 µg/mL) [10], guaiol (1.9%, IC_50Promastigote_ = 14 µg/mL and IC_50Amastigote_= 0.01 µg/mL) [54], caryophyllene oxide (0.88%, IC_50Promastigote_= 4.9 µg/mL and IC_50Amastigote_ = 4.4 µg/mL) [55], and *E*-caryophyllene (0.66%, IC_50Promastigote_= 49.9 µg/mL and IC_50Amastigote_ = 10.7 µg/mL) [14]. The observed activity of CLS-EO could probably be attributed to the minor components and/or synergistic action.

EOs and their constituents can act on parasites of the genus *Leishmania* in several ways: (i) affecting the layers of polysaccharides, fatty acids, and phospholipids in the autophagosomal structures, cytoplasmatic/mitochondrial/nuclear membranes, and chromatin; (ii) interrupting specific metabolic pathways for lipids and proteins; or (iii) increasing reactive oxygen species that cause DNA damage that leads to parasite death through necrosis or apoptosis [56].

The determination of the cytotoxicity is important to evaluate the selectivity of natural products as future antiprotozoal candidates. In this study, a low selectivity of CLS-EO was revealed. In contrast, the EO from leaves presented a better selectivity (SI > 5) [28]. EOs obtained from four *Croton* spp. showed lower cytotoxicity on the monocytic cell line AMJ2-C11 than the reference drug at 100 μg/mL [49]. *C. cajucara* EO did not display toxicity against mouse peritoneal macrophages at concentrations up to 500 µg/mL [20], while oils extracted from *Croton pallidulus* Baill., *Croton ericoides* Baill., and *Croton isabelli* Baill. showed significant cytotoxicities on the Vero cell line [57].

Nevertheless, the in vitro cytotoxicity of CLS-EO could be corroborated in animal models due to the more complex in vivo situation; a metabolic transformation of toxic molecules to nontoxic ones in multicellular organisms may occur, and thus toxicity might dramatically change under these conditions [58].

A biomolecular target is considered to be a protein or nucleic acid with biological activity (an enzyme, receptor, transcription factor, ion channel, transport protein, protein–protein interface, or ribonucleic acid (RNA)) that is linked to a disease or infection and can be modified by a small molecule or drug [59]. The primary criterion for a biological macromolecule (protein/enzyme/nucleic acid) being a target is that it should be essential for the survival of the organism or pathogen [60]. Several metabolic pathways are currently under study, including the metabolism of fatty acids, sterols, glucose, glycolipids, etc. [61]. Enzymes that play a role in the metabolic pathway of parasites are considered potential targets for the development of new antiparasitic compounds, including targets of the trypanothione, sterol, and polyamine biosynthetic pathways of *Leishmania* [62].

Low binding free energies were obtained for several of the evaluated compounds from CLS-EO by docking studies with Cyp51, TryR, TryS, and ArgI *Leishmania* enzyme targets. Our in silico molecular docking study was consistent with the previous experimental results regarding the low activity of the main CLS-EO compound (1,8-cineole). This directed us to more deeply explore the potential interactions and the binding profile with these enzymes of *epi*-*γ*-eudesmol, the most potentially active among the main CLS-EO compounds.

In *Leishmania*, polyamines are important molecules that possess antioxidant activity and are possibly involved in controlling reactive oxygen species induced apoptosis [63]. Polyamine biosynthesis enzymes are promising drug targets for the treatment of leishmaniasis, Chagas disease, and African sleeping sickness. Arginase (ArgI), which is a metallohydrolase, is the first enzyme involved in polyamine biosynthesis, and it converts arginine into ornithine and urea. Ornithine is used in the polyamine pathway that is essential for cell proliferation and ROS detoxification by trypanothione. In *Leishmania* species, arginase regulates parasite growth, differentiation, and infectivity [64]. Other studies demonstrated that coordination with the Mn^2+^ ion in the active binding site of ArgI is important to inhibit this target [65]. Other favorable interactions of different types were formed, such as Van der Waals and π-alkyl interactions with 12 residues (Ser150, Asn143, Ala140, Ile142, Glu197, Gly155, Glu288, Asp245, Asp137, Thr257, His139, His154, and His114) in the active site [66]. That is why those interactions were monitored for *epi*-*γ*-eudesmol as the most promising of the main CLS-EO compounds.

The enzyme TryR participates in polyamine-dependent redox metabolism and performs antioxidant functions to protect the parasite against oxidative damage [67]. The bifunctional TryS catalyzes the biosynthesis and hydrolysis of the glutathione-spermidine adduct trypanothione, the principal intracellular thiol-redox metabolite in parasitic trypanosomatids [34].

The TryR-ligand complex (*epi*-*γ*-eudesmol) showed hydrogen bonds through the hydroxyl group in the ligand and the Tyr198 residue in the enzyme (Figure 5), and hydrophobic interactions, mainly van der Waals interactions, with the residues Glu202, Met333, Leu334, Cys52, Thr335, Thr51, Arg287, Cys57, Gly56, Val55, Ser178, Phe203, and Phe182 involved in the active site [35]. All those interactions favor the TryR-ligand complex (*epi*-*γ*-eudesmol) stability and as a consequence increase its possibility to show activity in future experimental studies.

In 2019, Feitosa et al. [68] identified the key residues responsible for the inhibitory process to be Thr335 and Thr51, mainly through hydrophobic interactions, analogous to the results obtained in this study. Other important interactions involve two amino acid residues, Cys52 and Cys57, reported by Baiocco et al. [35] as amino acids that participate in the redox reactions of this enzyme and maintain a hydrophobic interaction with *epi*-*γ*-eudesmol.

Another key enzyme of *Leishmania* spp. is sterol 14α demethylase (Cyp51), which catalyzes the removal of the 14α-methyl group from precursors during ergosterol biosynthesis [69]. Unlike mammals, which can accumulate cholesterol from the diet, the blocking of ergosterol production in fungi and protozoa is lethal; it affects cytokinesis, stops cell growth, and eventually leads to the collapse of the cellular membrane [70].

Molecular docking studies with enzyme Cyp51 confirm that most of the interactions are of a hydrophobic nature. The interactions were found to be in accordance with those reported by Hargrove et al. [36] and Sheng et al. [71], who demonstrated that the interactions with the enzyme Cyp51 fundamentally occur by means of der van Waals interactions and π-π stacking.

Even though the chemical composition studies revealed that 1,8-cineole is the metabolite with the highest concentration in CLS-EO, the molecular docking studies showed that *epi*-*γ*-eudesmol, despite having a lower concentration, could be responsible for the antileishmanial activity. In addition, *epi*-*γ*-eudesmol had relatively lower binding energies compared to the antileishmanial control drugs, and the main interactions with the studied enzymes were found to be of a different nature, including van der Waals, hydrogen bonds, π-σ, π-alkyl, etc. This molecular docking study suggests that it is not necessarily the metabolite with the highest concentration that is responsible for the antiprotozoal activity. However, it provides important indications regarding the possible mechanism of action of the main metabolites present in CLS-EO and on which enzymes they might be acting. Further investigations with respect to the main components identified in CLS-EO should be conducted to better understand the possible mechanisms of action against *Leishmania* spp.

In addition, the lipophilic nature of EOs permits easy diffusion through cell membranes, and they may then act directly on the parasite or stimulate cellular mechanisms for its elimination. One of these mechanisms is mediated by a significant increase in nitric oxide (NO) production in infected macrophages, which together with reactive oxygen species participates in the destruction of phagocytosed microbes [62]. Some components present in CLS-EO showed activity against amastigotes of *Leishmania* spp. The monoterpene linalool (3.91%), isolated from the leaves of *C. cajucara,* exhibited its action through the mechanism described above [20], and *α*-pinene (11.05%) purified from the essential oil of *Syzygium cumini* L. produced an increase in NO levels as well as immunomodulatory activity by increasing phagocytic and lysosomal activity [10].

## 4. Materials and Methods

### 4.1. Plant Material

Fresh aerial parts of *C. linearis* were collected in the Siboney-Juticí Ecological Reserve, Santiago de Cuba, Cuba (at 19.958488 N, −75.692820 W) in September 2020. A voucher specimen was deposited in the Herbarium “Jorge Sierra Calzado” of the Eastern Center of Ecosystems and Biodiversity (BIOECO, Santiago de Cuba, Cuba) under registration 21 659 after authentication by the botanist Ing. Felix Acosta Cantillo.

### 4.2. Extraction and Analysis of the Essential Oil

The EO was extracted from fresh stems of *C. linearis* (CLS-EO) by a hydrodistillation–cohobation method in a classic Clevenger-type apparatus, using 400 g of the sample and 1.6 L of distilled water for 3 h. Then, the EO was collected manually and dried with anhydrous sodium sulfate (Sigma-Aldrich, St. Louis, MO, USA). CLS-EO was stored in an amber bottle and kept in a refrigerator at 4 °C until analysis. The yield was calculated and expressed in % (*v*/*w*). The chemical composition was determined by gas chromatography–mass spectrometry (GC–MS) using a Shimadzu GCMS-QP2010 Ultra system (Unit of Characterization and Analysis, Institute of Research of Drugs and Medicines, Federal University of Paraíba/Brazil) with an RTX-5MS capillary column (30 m × 0.25 mm × 0.25 µm). Helium was used as a gas carrier with a flow rate of 0.6 mL/min. The injection volume was 1 μL, with a split ratio of 100:1. The programmed temperature was set to 30 °C for 1 min, an increase of 40 °C/min until 140 °C, and an increase of 4 °C/min up to 300 °C. The mass spectra were recorded over a 60–260 amu range with 70 eV of ionization energy. The identification of the chemical constituents was carried out by a comparison with software libraries (NIST08 and FFNSC 1.3) and the calculated Kovats retention indices. The composition in percentage was calculated using the peak normalization method.

### 4.3. Antileishmanial Assay

#### 4.3.1. Parasites

The standard strain MHOM/77BR/LTB0016 of *L. amazonensis* was supplied by the Institute of Tropical Medicine “Pedro Kourí”, Havana, Cuba. The parasites were routinely isolated from BALB/c mouse lesions and were maintained as promastigotes in Schneider’s Insect Medium (Sigma, St. Louis, MO, USA) supplemented with 10% fetal bovine serum (FBS, Sigma, St. Louis, MO, USA) and antibiotics (100 μg of streptomycin per milliliter and 100 U of penicillin per milliliter, Sigma, St. Louis, MO, USA) at 26 °C.

#### 4.3.2. Antipromastigote Assay

A stock solution of CLS-EO was diluted in 100% DMSO at 20 mg/mL. The assay was carried out under the same methodology reported previously [28]. Serial 1:2 dilutions were carried out to obtain final concentrations between 12.5 and 200 µg/mL, and parasites (2 × 10^5^ promastigotes/mL) were treated for 72 h at 26 °C. The cellular viability was determined by a colorimetric assay with 20 µL of 3-[4,5-dimethylthiazol-2-yl]2,5-diphenyltetrazolium bromide (MTT; SIGMA, St. Louis, MO, USA). After 4 h of incubation, the supernatant was eliminated, tetrazolium salt was dissolved with 100 µL of DMSO, and the microplate was read in an ELISA microplate reader (Sirio S Reader, 2.4-0, Italy) at 540 nm and 620 nm as reference wavelengths [72]. The median inhibitory concentration (IC_50_) was determined by a dose–response linear regression analysis. Each experiment was performed in duplicate, and the results were expressed as means ± standard deviations.

#### 4.3.3. Antiamastigote Assay

Peritoneal macrophages from BALB/c mice were collected and seeded with a density of 10^6^/mL in 24-well plates and incubated at 37 °C and 5% CO_2_ atmosphere for 2 h. Nonadherent cells were removed. Cells were infected with promastigotes (in the stationary phase) at a parasite/macrophage ratio of 4:1 and incubated for 4 h under the same conditions. Afterwards, the assay was carried out under the same methodology reported previously [28]. Serial 1:2 dilutions were performed to test concentrations between 12.5 and 100 µg/mL and were incubated for 48 h. Then, the supernatant was removed, and monocultures were fixed with methanol and stained with Giemsa. For each sample, the number of intracellular amastigotes and the percentage of infected macrophages were determined in 25 macrophages by counting at a microscope at 100× under immersion oil. The results were expressed as the percentage of reduction of the infection rate (which was obtained by multiplying the percentage of infected macrophages by the number of amastigotes per infected macrophage) in comparison with the negative controls. The IC_50_ was determined by a dose–response linear regression analysis. Each experiment was performed in duplicate, and the results were expressed as means ± standard deviations. In both experiments, Pentamidine^®^ (Richet, Buenos Aires, Argentina) at 10 mg/mL was used as a reference drug.

#### 4.3.4. Cytotoxic Assay

The median cytotoxic concentration (CC_50_) was determined on peritoneal macrophages from BALB/c mice, which were collected and washed with RPMI 1640 medium (SIGMA) supplemented with antibiotics (200 IU of penicillin and 200 µg of streptomycin per milliliter). The assay was carried out under the same methodology as reported previously [28]. The cellular viability was determined by a colorimetric assay with MTT, as previously described, but using 15 µL of MTT solution/well. The CC_50_ was determined by a dose–response linear regression analysis. Each experiment was performed in duplicate, and the results were expressed as means ± standard deviations. The selectivity index (SI) was calculated by the following formula: CC_50_/IC_50_.

### 4.4. Molecular Docking Studies

Molecular modeling was performed using the high-performance computing capabilities of the cluster of the *Universidad de Oriente*, Cuba (HPC-UO) (https://portal.uo.hpc.cu/website/ (accessed on 6 November 2022)). Molecular docking studies of the CLS-EO metabolites against *Leishmania* target proteins were performed using AutoDock 4.2 software [73]. The 3D structures of the main compounds identified in the study (Figure 2) were constructed using the programs included in the ChemOffice 17.1 software [74].

The crystal structures of the target proteins were obtained from the Protein Data Bank (PDB ID) with the codes sterol 14α demethylase (3L4D), trypanothione reductase (2JK6), trypanothione synthetase (2VOB), and arginase I (4ITY), which are considered relevant and interesting molecular targets in *Leishmania* spp. [75].

The assay parameter was the Lamarckian genetic algorithm (GA) with the following conditions: population amount, 100; maximum number of evals, 2,500,000; with maximum number of generations, 27,000; and other parameters were taken by default. The induced coupling geometric regions were determined with AutoDockTools 1.5.6 [73]. AutoDock requires precalculated grid maps, one for each atom type present in the ligand being docked, as it stores the potential energy arising from the interaction with the macromolecule. This grid must surround the region of interest (active site) in the macromolecule (Table 4).

The docked conformations of each ligand were ranked into clusters based on the binding energy. During the molecular docking, interactions (ΔG_dock_ = ΔG_vdW_ + ΔG_elec_ + ΔG_hbond_ + ΔG_desolv_ +ΔG_tors_) were evaluated by molecular mechanics (MM) in AutoDock4 [71]. The top ranked conformations were visually analyzed; Discovery Studio Visualizer v.20.1.0.19295 [76] was used to plot the bonding and nonbonding interactions of the ligand with the receptor in the receptor–ligand complex.

Docking calculations were validated by redocking the co-crystallized ligands in the receptor structures [77]. However, in some target structures, no inhibitors were present and the known antileishmanial drugs pentamidine [78] and miltefosine [79], taken from DrugBank, were used. They were built and docked. Due to the high molecular weights of the control compounds, we accounted for the recognized bias and homogenized the energy value using the formula DS_norm_ = 5.71 × ΔG_dock_/MW^1/3^, where DS_norm_ is the normalized docking score, ΔG_dock_ is the docking energy from the molecular docking program, and 5.71 is a scaling constant to make the average DS_norm_ values comparable to ΔG_dock_ [80].

## 5. Conclusions

This study is the first report of the chemical composition and antileishmanial evaluation of CLS-EO. The chemical composition of this oil revealed high concentrations of monoterpene hydrocarbons and oxygenated monoterpenes, with 1,8-cineole as the main compound. CLS-EO showed activity on the promastigote and amastigote forms of *L. amazonensis,* with low values of SI. The biological activity can be attributed to the synergistic interactions of the EO components. A molecular docking analysis confirmed the stability of the complexes between *epi*-*γ*-eudesmol and the different target enzymes of *Leishmania*, which implies that the antileishmanial action could occur by different mechanisms of action whose main component would be this metabolite.

In conclusion, these findings complete a series of studies about the pharmacological potentialities of *C. linearis* essential oils. In addition, this study provides support for the further exploration of the main components from CLS-EO as antileishmanial agents and may contribute to research of new candidates for this NTD.

## Figures and Tables

**Table 1:** The chemical composition of the essential oil from *Croton linearis* stems (CLS-EO) growing in the Siboney-Juticí Ecological Reserve, Santiago de Cuba.

Rt ^a^ (min)	Compounds	% RA ^b^	RI_exp_^c^	RI_lit_^d^	Rt (min)	Compounds	% RA	RI_exp_	RI_lit_
5.73	*α*-thujene	1.28	926	926	25.86	not identified	0.32	1427	-
5.94	***α*-pinene**	**11.05**	934	938	27.18	*α*-humulene	0.18	1458	1454
6.37	*α*-fenchene	0.45	949	943	28.30	*α*-amorphene	0.38	1484	1484
7.07	***cis*-sabinene**	**8.06**	974	976	28.75	*β*-selinene	0.47	1495	1497
7.19	*β*-pinene	1.18	978	979	28.93	*α*-selinene	0.23	1499	1499
7.55	*β*-myrcene	1.30	991	990	29.41	*γ*-cadinene	0.19	1511	1512
8.05	*α*-phellandrene	1.20	1006	1002	29.80	cubebol	0.14	1521	1521
8.46	*α*-terpinene	0.85	1017	1017	30.10	zonarene	0.61	1528	1528
8.75	***p*-cymene**	**5.72**	1025	1026	30.70	selina-4(15),7(11)-diene	0.57	1542	1540
8.90	limonene	1.31	1029	1031	31.19	germacrene B	0.27	1555	1555
9.00	**1,8-cineole (eucalyptol)**	**27.83**	1032	1033	31.68	dihydroisocaryophyllene epoxide	0.89	1566	1565
9.17	*Z*-*β*-ocimene	1.04	1036	1041	32.08	germacrene D-4-ol	0.17	1576	1576
9.56	*E*-*β*-ocimene	0.19	1046	1044	32.28	spathulenol	0.16	1581	1578
10.01	*γ*-terpinene	1.00	1058	1059	32.58	caryophyllene oxide	0.88	1589	1589
10.36	*cis*-sabinene hydrate	0.21	1068	1066	33.25	guaiol	1.93	1605	1605
10.53	*cis*-linalool oxide	0.13	1072	1074	33.41	copaborneol	0.14	1609	1593
11.17	terpinolene	0.29	1089	1088	33.64	humulene epoxide II	0.14	1615	1614
11.63	**linalool**	**3.91**	1101	1098	33.88	(2*Z*)-2,6-dimethyl-2,7-octadiene-1,6-diol	0.54	1621	1617
12.57	*cis*-*p*-menth-2-en-1-ol	0.11	1123	1121	34.04	***epi*-*γ*-eudesmol**	**4.15**	1626	1627
13.35	*cis*-verbenol	0.14	1141	1142	34.38	*γ*-eudesmol	0.35	1634	1635
14.56	*δ*-terpineol	0,33	1170	1173	34.60	3-methyl-5-(1,4,4-trimethylcyclohex-2-enyl)pentan-1-ol	2.09	1640	1637
15.00	**terpinen-4-ol**	**2.55**	1180	1177	34.80	hinesol	2.98	1645	1638
15.61	***α*-terpineol**	**4.35**	1194	1189	34.88	*α*-muurolol	1.49	1649	1649
17.50	thymol methyl ether	0.78	1237	1234	35.16	valerianol	1.15	1654	1655
17.91	2-isopropyl-1-methoxy-4-methylbenzene	0.18	1246	1244	35.42	cadin-4-en-10-ol	0.88	1660	1663
19.79	isobornyl acetate	0.45	1289	1286	36.10	9*E*,12*Z*-tetradecadien-1-ol	0.23	1677	1676
24.50	*β*-elemene	0.66	1395	1394	36.85	geranyl tiglate	0.79	1697	1700
25.25	longifolene	0.28	1412	1412	37.04	*E,E*-farnesal	0.19	1729	1730
25.71	*E*-caryophyllene	0.66	1423	1427					
Monoterpene hydrocarbons						14 (34.84%)			
Oxygenated monoterpenes						12 (41.05%)			
Sesquiterpene hydrocarbons						12 (4.82%)			
Oxygenated sesquiterpenes						19 (19.29%)			

^a^ RTmin: Retention time; ^b^ %RA: Relative abundance; ^c^ RI_exp_: Retention index relative to *n*-alkanes (C_8_–C_20_) on the RTX-5MS column; ^d^ RI_lit_: Kovats retention index (values from the literature). The main compounds are highlighted in **bold font**.

**Table 2:** The antileishmanial activity, cytotoxic effect, and selectivity index of the essential oil of stems from *Croton linearis* growing in Santiago de Cuba.

Sample	Cytotoxicity	Anti-*Leishmania* Activity			
	Macrophages CC_50%_^a^ ± SD ^b^ (µg/mL)	Promastigotes IC_50%_^c^ ± SD (µg/mL)	Selectivity Index ^d^	Amastigotes IC_50%_ ± SD (µg/mL)	Selectivity Index ^e^
CLS-EO	49.0 ± 5.0	21.4 ± 0.1	2	18.9 ± 0.3	3
Pentamidine^® f^	11.7 ± 1.7	0.4 ± 0.1	29	1.3 ± 0.1	9

^a^: Median cytotoxic concentration; ^b^: Standard deviation; ^c^: Median inhibitory concentration; ^d^: CC_50_/IC_50%Promastigote_; ^e^: CC_50_/IC_50%Amastigote_; ^f^ Reference drug.

**Table 3:** Docking scores (ΔG_dock_) and normalized docking scores (DS_norm_) (kcal/mol) for the main compounds of essential oil from *Croton linearis* and the target enzymes chosen for *Leishmania*.

Compounds	MW (g/mol)	*Leishmania* Enzyme Targets							
		Cyp51		TryR		TryS		ArgI	
		ΔG_dock_	DS_norm_	ΔG_dock_	DS_norm_	ΔG_dock_	DS_norm_	ΔG_dock_	DS_norm_
1,8-cineole	154.253	−5.84	−6.22	−5.13	−5.46	−5.57	−5.93	−4.47	−4.76
α-pinene	136.238	−5.63	−6.25	−4.89	−5.43	−5.85	−6.49	−4.1	−4.55
*α*-terpineol	154.253	−5.88	−6.26	−5.72	−6.09	−7.09	−7.55	−6.54	−6.96
*cis*-sabinene	136.238	−5.27	−5.85	−4.93	−5.47	−5.78	−6.42	−3.83	−4.25
*epi*-*γ*-eudesmol	222.372	−7.79	−7.34	−7.20	−6.79	−7.87	−7.42	−7.87	−7.42
linalool	154.253	−5.49	−5.85	−4.84	−5.15	−6.44	−6.86	−6.28	−6.69
*p*-cymene	134.222	−5.28	−5.89	−4.88	−5.44	−6.18	−6.89	−4.01	−4.47
terpinen-4-ol	154.253	−5.73	−6.10	−5.48	−5.84	−6.35	−6.76	−5.4	−5.75
Miltefosine ^a^	407.576	−8.96	−6.90	−4.72	−3.64	−5.47	−4.21	−2.99	−2.30
Pentamidine ^a^	340.427	−9.22	−7.54	−8.57	−7.01	−8.71	−7.12	−6.00	−4.91

MW: Molecular weight; ^a^: control drugs used for molecular docking.

**Table 4:** Grid box parameters selected for the target enzymes.

Protein	Species	PDB ID	Resolution	Grid Box Center Coordinates			Grid Box Size
				x	y	z	
Cyp51	*L. infantum*	3L4D	2.75	31.917	−28.96	−1.658	50 × 50 × 50
TryR	*L. infantum*	2JK6	2.95	30.449	47.483	−4.312	
TryS	*L. major*	2VOB	2.3	−5.339	−21.67	8.498	
ArgI	*L. mexicana*	4ITY	1.8	15.141	−15.125	−5.4	

Cyp51: sterol 14α demethylase, TryR: trypanothione reductase, TryS: trypanothione synthetase, ArgI: arginase I. Units for the coordinates and box size are Å.

## Data Availability

Not applicable.

## Supplementary Materials

The following supporting information can be downloaded at: https://www.mdpi.com/article/10.3390/antibiotics11121712/s1, Figure S1: Mass spectra of main essential oil components from *Croton linearis* stems: **A**→ 1,8-cineole, **B**→ *α*-pinene, **C**→ cis-sabinene, **D**→ *p*-cymene, **E**→ *α*-terpineol, **F**→ *epi*-*γ*-eudesmol, **G**→ linalool, **H**→ terpinen-4-ol.
