# Supplementary material for: Chemical Composition and In Vitro and In Silico Antileishmanial Evaluation of the Essential Oil from Croton linearis Jacq. Stems
Source: Antibiotics (Basel). 2022 Nov 28;11(12):1712. doi: 10.3390/antibiotics11121712 (PMC9774621; doi:10.3390/antibiotics11121712)
Supplement: Supplementary file 1 [file antibiotics-11-01712-s001.zip › antibiotics-2039180-supplementary.pdf]

Supplementary Material

Chemical composition, *in vitro* and *in silico* antileishmanial evaluation of the essential oil from *Croton linearis* Jacq. stems.

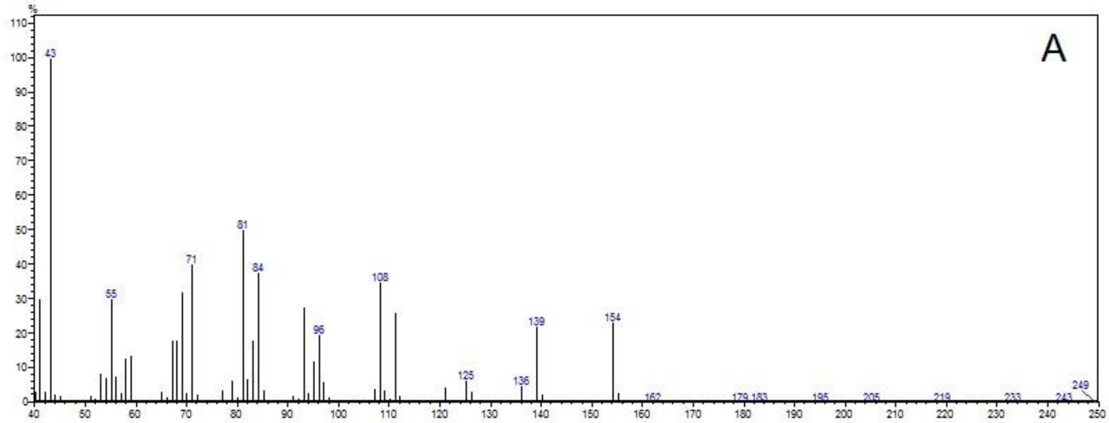

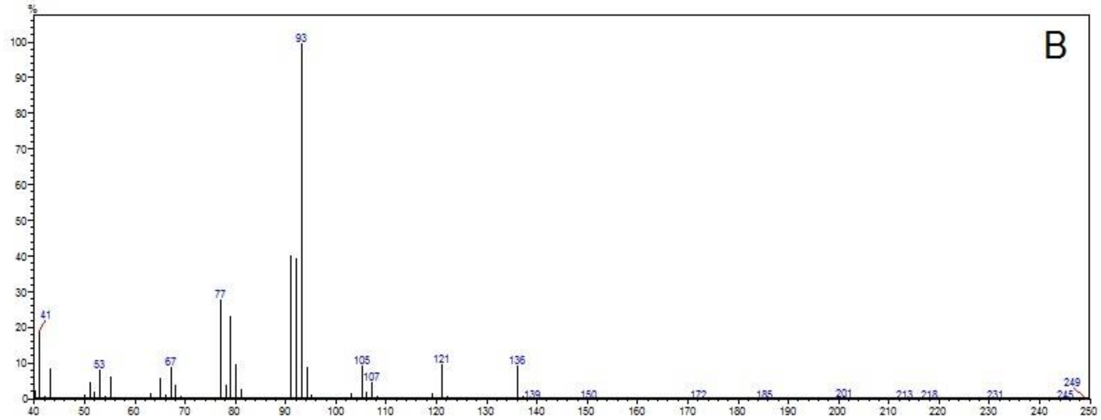

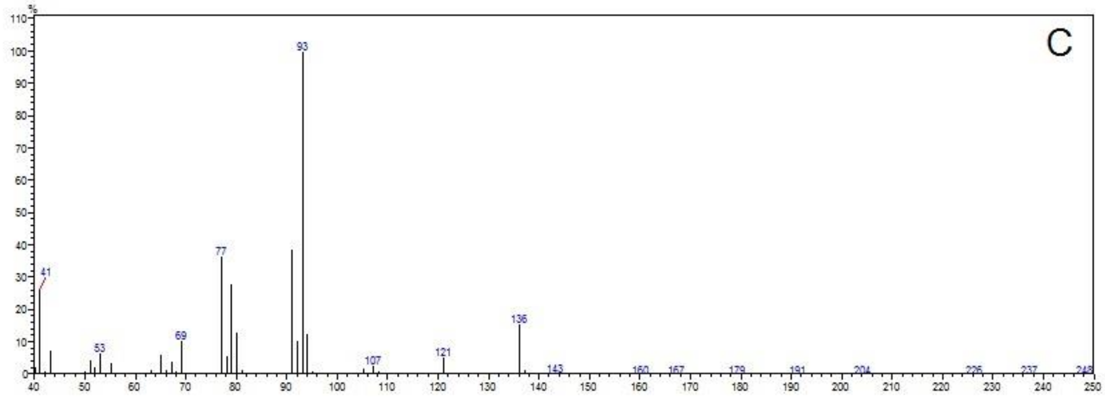

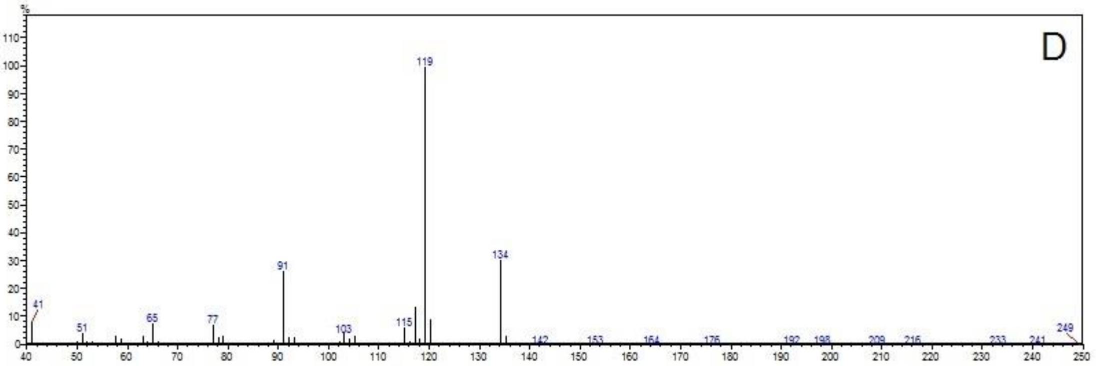
D
%
119
134
91
41
51
65
77
103
115

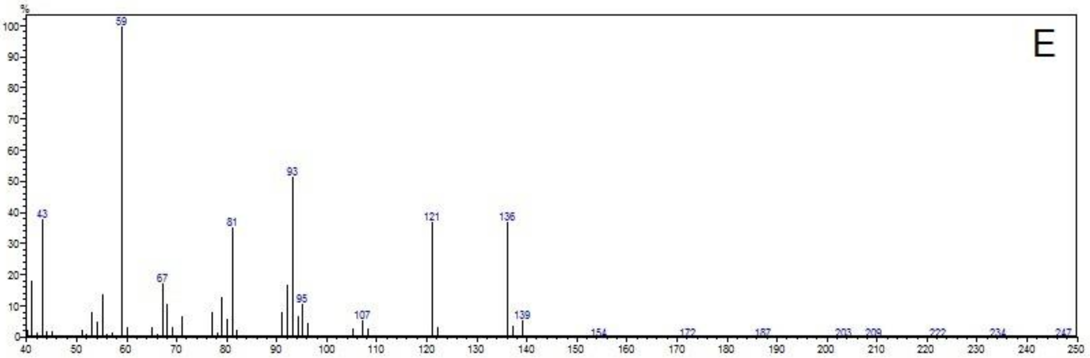
E
%
59
93
43
81
121
136
67
95
107
139

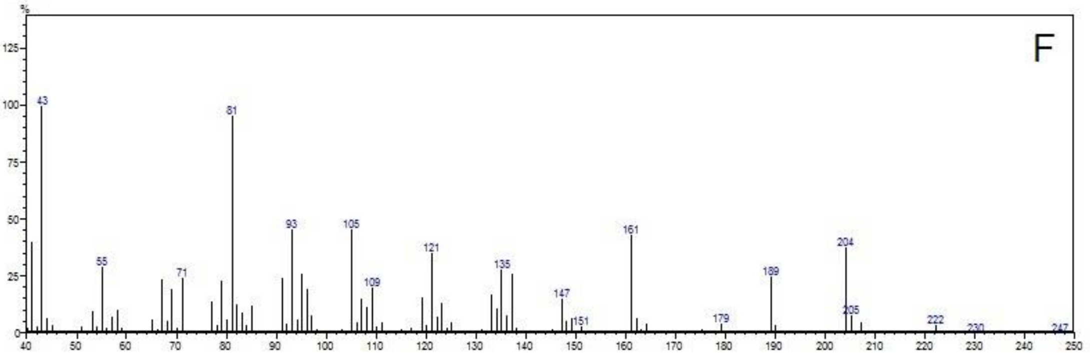
F
%
43
81
93
105
161
204
121
55
135
71
109
189
147
205
179
151
222

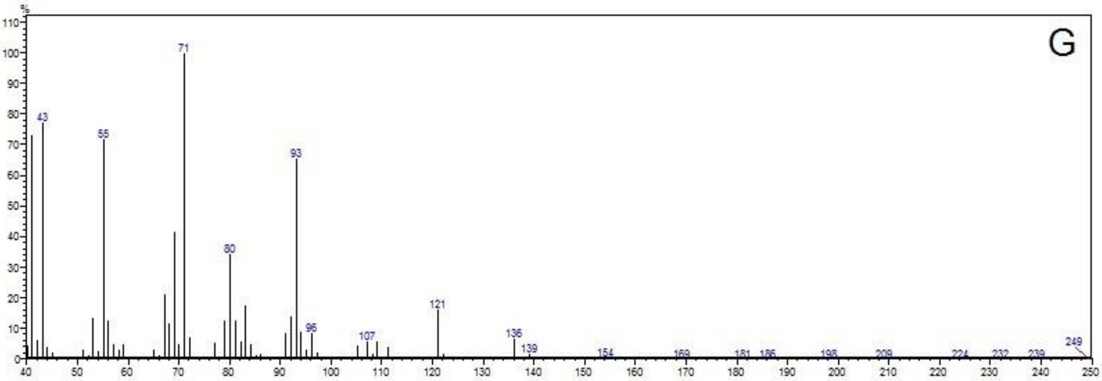
G
%
71
43
55
93
80
121
96
107
136
139

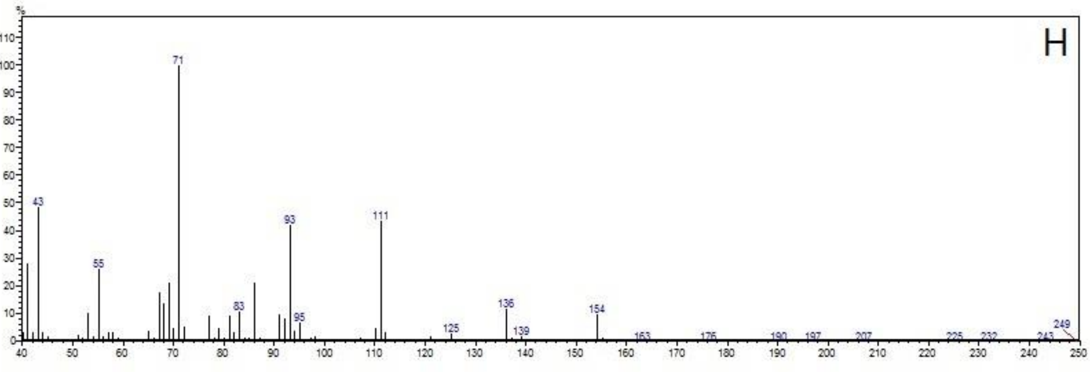

Figure S1. Mass spectra of main essential oil components from *Croton linearis* stems: **A**→ 1,8-cineole, **B**→ *α*-pinene, **C**→ *cis*-sabinene, **D**→ *p*-cymene, **E**→ *α*-terpineol, **F**→ *epi*-*γ*-eudesmol, **G**→ linalool, **H**→ terpinen-4-ol.
